# Case studies emphasising the difficulties in the diagnosis and management of alveolar echinococcosis in rural China

**DOI:** 10.1186/1756-3305-4-196

**Published:** 2011-10-09

**Authors:** Donald P McManus, Zhengzhi Li, Shukun Yang, Darren J Gray, Yu Rong Yang

**Affiliations:** 1 Queensland Institute of Medical Research, Herston, Brisbane, Australia; 2 Ningxia Medical University, Yinchuan, Ningxia, The People's Republic of China; 3 Yinchuan First People's Hospital, Yinchuan, Ningxia, The People's Republic of China; 4 Griffith Health Institute, Griffith University, Meadowbrook, Australia

**Keywords:** Alveolar echinococcosis, Ningxia Hui Autonomous Region, The People's Republic of China, metastatic lesions in the brain; diagnosis, albendazole treatment

## Abstract

**Background:**

Human alveolar echinococcosis (AE) is caused by the accidental ingestion of the eggs of the fox tapeworm *Echinococcus multilocularis*. AE occurs frequently in rural western China due to the poor levels of hygiene, the close contact of people with dogs, and the lack of appropriate facilities for the correct and rapid diagnosis of the disease.

**Findings:**

We describe a case of a patient with hepatic AE, and AE metastases of the brain. She was mistakenly diagnosed with suspected undifferentiated metastatic cancer of the liver and brain, and with a pulmonary bacterial infection, but was subsequently correctly diagnosed during a follow-up field survey for echinococcosis. The diagnosis of brain AE was confirmed by pathological examination of tissue biopsies removed during neurosurgery. We also briefly describe other symptomatic and asymptomatic AE cases, identified by chance, likely due to the inadequate facilities available in rural communities in China for AE diagnosis and management, since the rapid and accurate diagnosis of metastatic AE requires a high level of expertise in the appropriate diagnostic procedures.

**Conclusions:**

This report highlights the necessity for an upgrade in the diagnosis, treatment, prevention and control of AE in rural China.

## 

Alveolar echinococcosis (AE) is caused by the larval stage of the fox tapeworm *Echinococcus multilocularis*. *E. multilocularis *has a sylvatic lifecycle with canids (foxes and coyotes) as the definitive hosts, and arvicolid rodents (voles), which harbour the larval metacestode stage, as intermediate hosts. Foci of human AE also occur where foxes or domestic dogs feed on infected rodents [1] and where there is accidental ingestion of *E. multilocularis *eggs from canine faecal matter.

Generally, human infection with *E. multilocularis *results in an asymptomatic chronic disease lasting 10-15 years. AE is characterised by primary lesions in the liver which produce a tumour-like multi-vesicular, infiltrating structure. Early diagnosis of AE by characteristic symptoms, imaging techniques, serology, histopathological examination or molecular analysis of biopsied material can improve the management and treatment of AE patients. Recently developed and improved serological tests for AE have been shown to be reliable [2] but, in most rural communities of China, these tests are not generally available. Consequently, failure to diagnose AE [1] or its misdiagnosis [3] leads to advanced disease making treatment difficult and prognosis poor.

Here we describe the diagnosis and management of a small series of AE patients, with or without symptoms, identified by chance in Ningxia Hui Autonomous Region (NHAR). Using these patients as examples, we illustrate the diagnostic and treatment challenges that AE poses in rural China as a consequence of the poor public health infrastructure currently available in this setting.

In 2006, a 46-year old Han Chinese female who had had an ingravescent headache for three months suddenly lost consciousness and was taken to Yinchuan Hospital in Yinchuan (the capital of NHAR), after being first admitted as an emergency case to a local rural medical centre in Xiji County in south NHAR. Head Magnetic Resonance Imaging (MRI) scans indicated the presence of lesions occupying the right frontal, temporal and occipital lobes of the brain (Figure 1a, b, c) with the largest lesion (3.5 × 3 cm) located in the temporal lobe (Figure 1a, b). A chest X-ray revealed multiple flake shadows on both sides of the patient's lung lobes (Figure 1d); an abdominal ultrasound scan showed an uneven dense lesion in the right liver with scattered calcification (Figure 1e). A full clinical examination and neurological assessment were then undertaken. Routine laboratory tests included blood cell and haematoblast counts and determination of haemoglobin levels and blood type.

**Figure 1:**
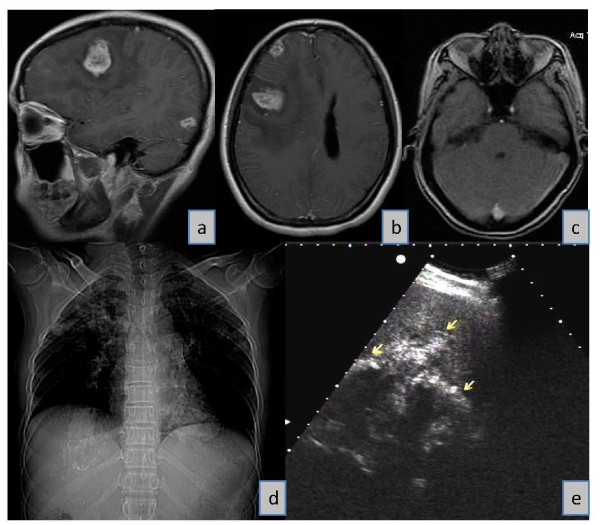
**Patient diagnostic images**: Magnetic Resonance Imaging (***Panels a, b, c***): ***Panel a ***is a sagittal T1 image of the brain showing AE lesions in the temporal (the largest lesion is 3.5 × 3 cm in size) and occipital lobes; ***Panel b ***is an Axial T1 image of the brain showing AE lesions in the frontal and temporal lobes; ***Panel c ***is an Axial T1 image of the brain (at a lower level than ***Panel b***) showing a lesion in the occipital lobe; ***Panel d ***is a chest x-ray image showing many cotton-flower shape shadows spreading on both sides of the lungs, but mainly involving the upper and middle lung areas; ***Panel e ***is an abdominal ultrasound scan showing a P4 AE lesion (arrowed) in the right liver lobe.

The primary diagnosis was of undifferentiated metastatic cancer in the liver and brain, with current pulmonary bacterial infection. Due to her poor finances, the patient was able to pay for basic treatment only and no further examination was undertaken to determine the source of the metastasis. She was discharged from hospital shortly thereafter. One month later, during a routine follow-up field survey for echinococcosis in Xiji County, her symptoms recurred and she was re-admitted to a local county hospital. Since the survey, undertaken by Ningxia Medical University, provided free clinical examination and all treatment costs for any suspected echinococcosis case, the local hospital physician and the relatives of the patient requested further investigation of her symptoms.

After a systematic review of her clinical records, it was suspected that the patient had hepatic AE which had metastasised to the brain and lungs. A 5-ml sample of venous blood was taken for serum IgG-ELISA using recombinant Em18 [2] to detect anti-*E. multilocularis *specific antibody and recombinant EgB [4] to detect anti-*E. granulosus *antibody. The serology was kindly undertaken in the laboratory of Professor Akira Ito (Asahikawa Medical College, Japan) and the outcome supported the diagnosis of active AE (Em18-antibody-positive but EgB-antibody-negative). Therefore, the local Xiji County hospital physician in charge of the patient was advised to provide her with albendazole (ABZ) (15 mg/kg per day). After 6 months treatment, the patient suffered no further headaches and she was able to walk. The patient then underwent surgery to remove the AE lesions from the brain, but, this proved unsuccessful as only a partial resection of the AE mass was possible. Histopathological examination of biopsy material obtained during the surgery confirmed the diagnosis of metastatic AE in the brain. The final diagnosis was confirmed as hepatic AE (Stage P4) with metastasis involving the lungs and brain (M1) and the patient was monitored annually thereafter.

Initially, the patient's symptoms were thought to be caused by an intra-cerebral disorder. The patient and her family were unaware of her true condition and had not sought medical advice before she lost consciousness leading to her admission to hospital as an emergency case. The poor health status of this patient from rural China is common, being a feature highlighted previously [5,6]. It results from the poor education available to residents of these rural communities, the prevailing underdeveloped economy, the inadequate medical/health infrastructure, and the general lack of medical insurance coverage for health care.

This patient presented initially without any apparent hepatic involvement despite the majority of the liver having advanced AE lesions (AE stage P4 N0 M1) [7,8]). Although unusual, other cases of AE without hepatic involvement have been reported [9]. Differential diagnosis for AE should generally include assessment for the presence of tumours and other lesions such as abscesses caused by TB [10], which may require specific pathological examination [3], or molecular analysis of biopsy material to provide a definitive diagnosis [11].

Although imaging and general clinical examination had been undertaken, this case was initially misdiagnosed. Importantly, the patient, being resident in a poor rural area, was unable to receive a definitive diagnosis in a better equipped hospital due to her poor financial status and the fact she had no medical insurance. The only option available to her was a local medical centre, where there is a general lack of facilities and an absence of well-trained health-workers to undertake the appropriate diagnosis [12]. It is noteworthy that, prior to the echinococcosis patient follow-up survey in Xiji County referred to above, specific serology for AE had been largely ignored in local NHAR hospitals, even in areas known to be hyper-endemic for echinococcosis. Unfortunately, the circumstances of this case are common, rather than rare [13], in rural China, where specific diagnostic methods for AE are not available, and the clinical findings are often misinterpreted as either due to cancer [3] or another infectious disease [14].

Patients from areas in China endemic for echinococcosis usually have to undergo repeated medical examination without the availability of resources for the specific diagnosis of the disease; this is neither time- nor cost-effective, and patient treatment is often delayed [15]. Moreover, the extensive haematogenous metastasis that often occurs in AE may complicate diagnosis, especially in resource-poor rural areas without the availability of the relevant diagnostic procedures [5,12]. Nevertheless, physical examination of the liver and abdominal area of a suspected AE patient, together with some knowledge of the local presence of echinococcosis, a clinical history of hepatic involvement, appropriate serology, and examination of biopsy materials should lead to a correct diagnosis. In this respect, a 31-year-old male, with extra-hepatic symptoms, who had felt fatigued for two years and who had gradual aggravated breathing and a dry cough, was first misdiagnosed with an unknown type of metastatic lung cancer after chest radiographic imaging at Xiji county hospital. Subsequently, a research team from Ningxia Medical University undertook an echinococcosis patient follow-up community survey involving ultrasound abdominal imaging and, with local physicians from Xiji county hospital, combined the ultrasound findings with the patient's clinical and epidemiological background data, to definitely diagnose a case of hepatic AE (Stage P4) with metastatic involvement of the lungs (AE P4 N0 M1).

Two additional cases of liver AE were also disclosed by chance. One was a 25-year-old male who was asymptomatic but was diagnosed with liver AE (Stage P4) when he accompanied his father during an echinococcosis patient follow-up examination by the Ningxia Medical University research team at Xiji county hospital. The other was a recently married 19-year-old female from Huoshizhai Township, Xiji County, whose husband had been diagnosed with liver AE (Stage P2) during an echinococcosis community survey 12 months previously. He was invited to attend a clinical follow-up locally and she accompanied him. Although having no symptoms, she was invited to undertake a routine clinical examination which revealed early stage (P1) AE in the left lobe of her liver.

We have previously described many AE cases diagnosed during community surveys in NHAR [13] or during patient follow-up [5,15], but AE case identification is rare in hospital records [6]. This reflects the grossly inaccurate hospital records for AE case detection, particularly in this under-developed rural region of China.

It has been previously reported that, if left untreated > 95% of AE patients will die within 10 years following diagnosis [16]. As described here, surgery can be used [5], even for late stage AE cases, but this may be problematic because of the difficulty in removing all of the parasitic lesions [3]. Therefore, chemotherapy should be used in combination with surgery to improve the survival of patients with incomplete or non-resectable AE. The beneficial effects of albendazole for treatment of AE are well known [17,18]. Indeed, ABZ is highly effective against AE and should be used before surgical intervention, together with regular patient follow-up post-treatment.

These cases emphasise the problems associated with late diagnosis of AE, a lack of awareness of the disease in rural communities [19], inadequate diagnostic facilities, and no specific clinical procedures available for examination of a disease which is a major public health problem in many rural and remote areas of western China [6]. There is an urgent need to enhance local health care infrastructures there to improve the expertise and quality of local health providers, a requirement for adequate facilities to screen populations for infection, and to educate rural communities in areas endemic for echinoccocosis about the disease and its prevention. The cost-effectiveness of diagnosis and treatment programs for AE should also be reviewed to further support rural health providers which include rural hospital workers and clinical practitioners.

## Competing interests

The authors declare that they have no competing interests.

## Authors' contributions

DPM and YRY conceived the study, YRY, ZL carried out the survey and questionnaire work, SKY undertook the MRI, CXR and US examinations, YRY, DJG and DPM analysed the data and DPM, YRY wrote the manuscript. All authors read and approved the final version of the manuscript.
